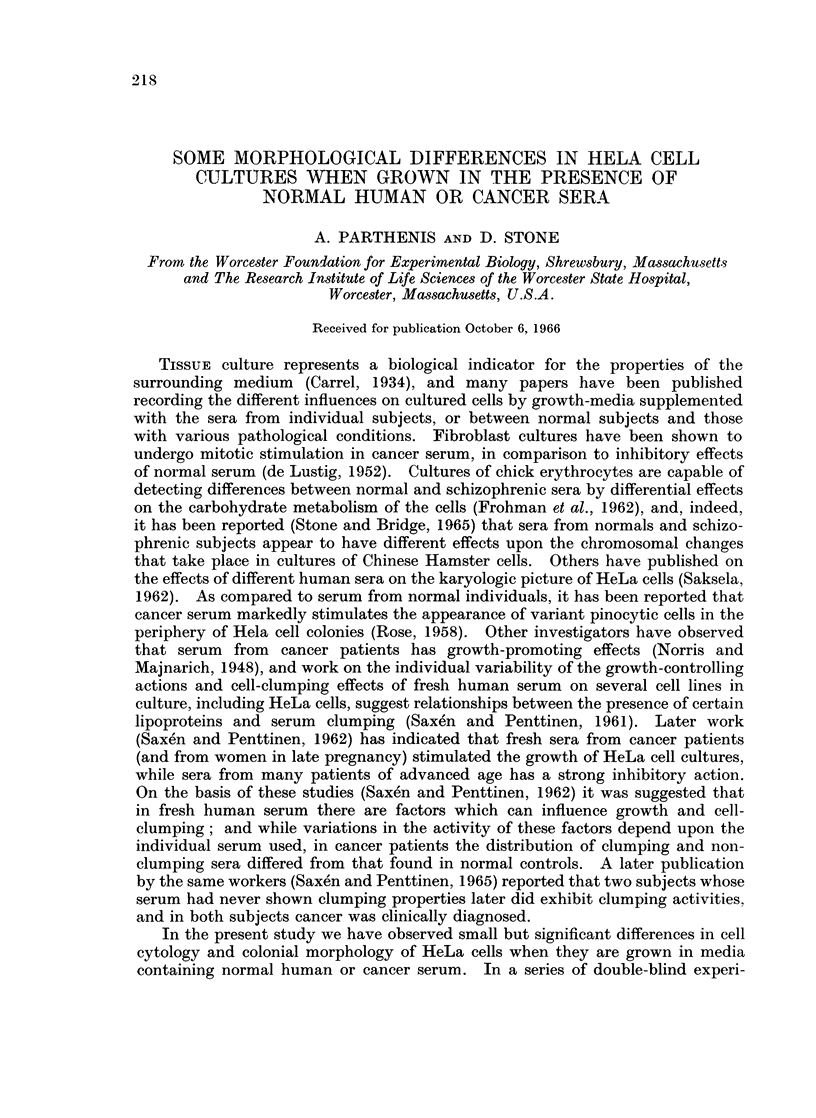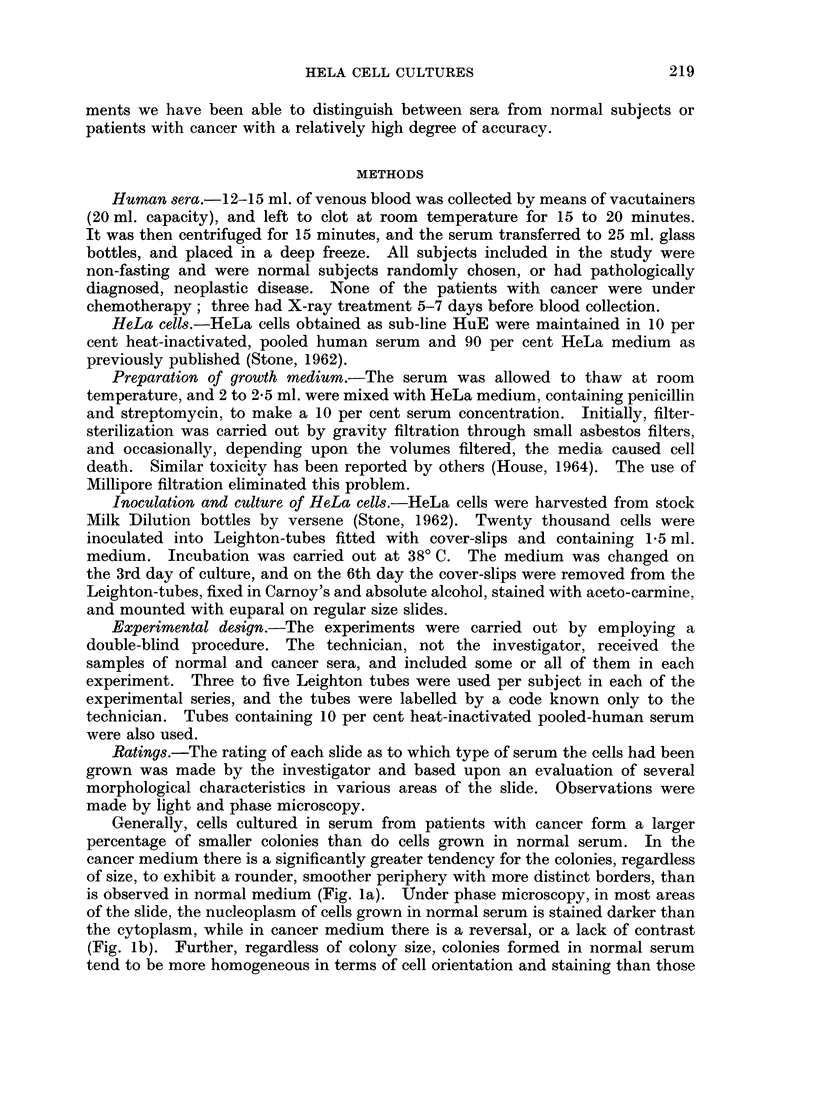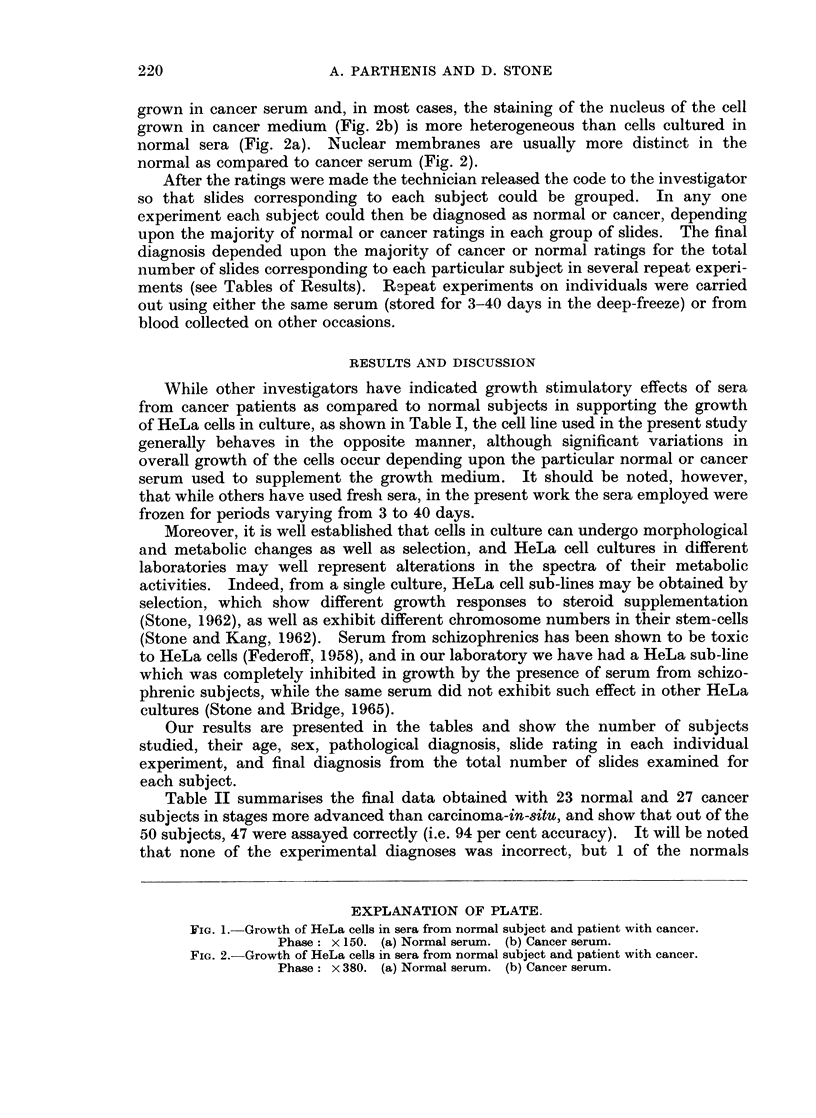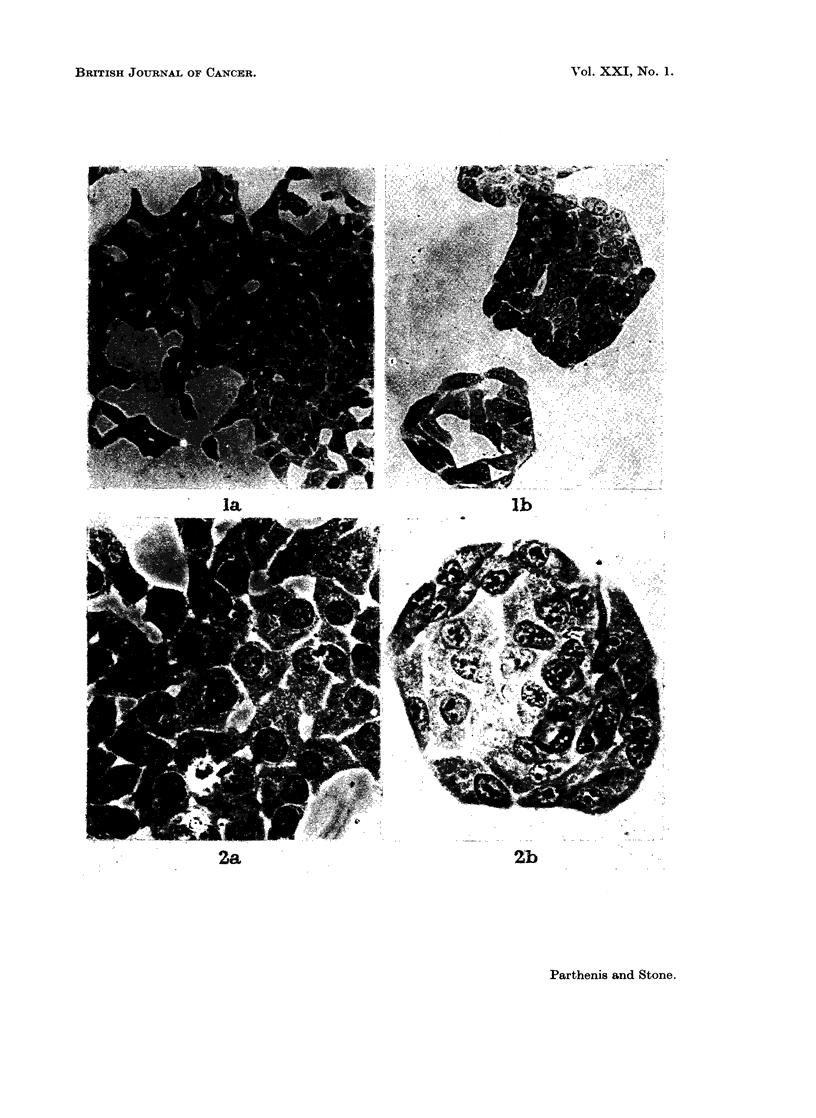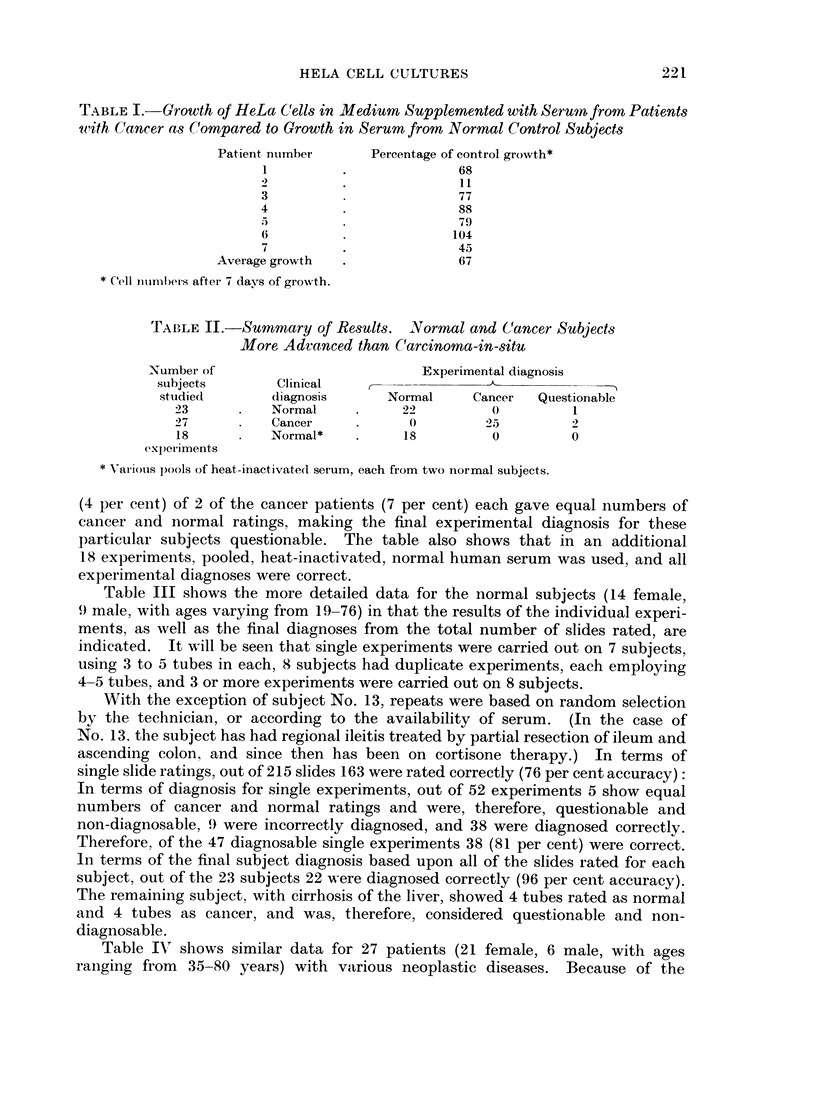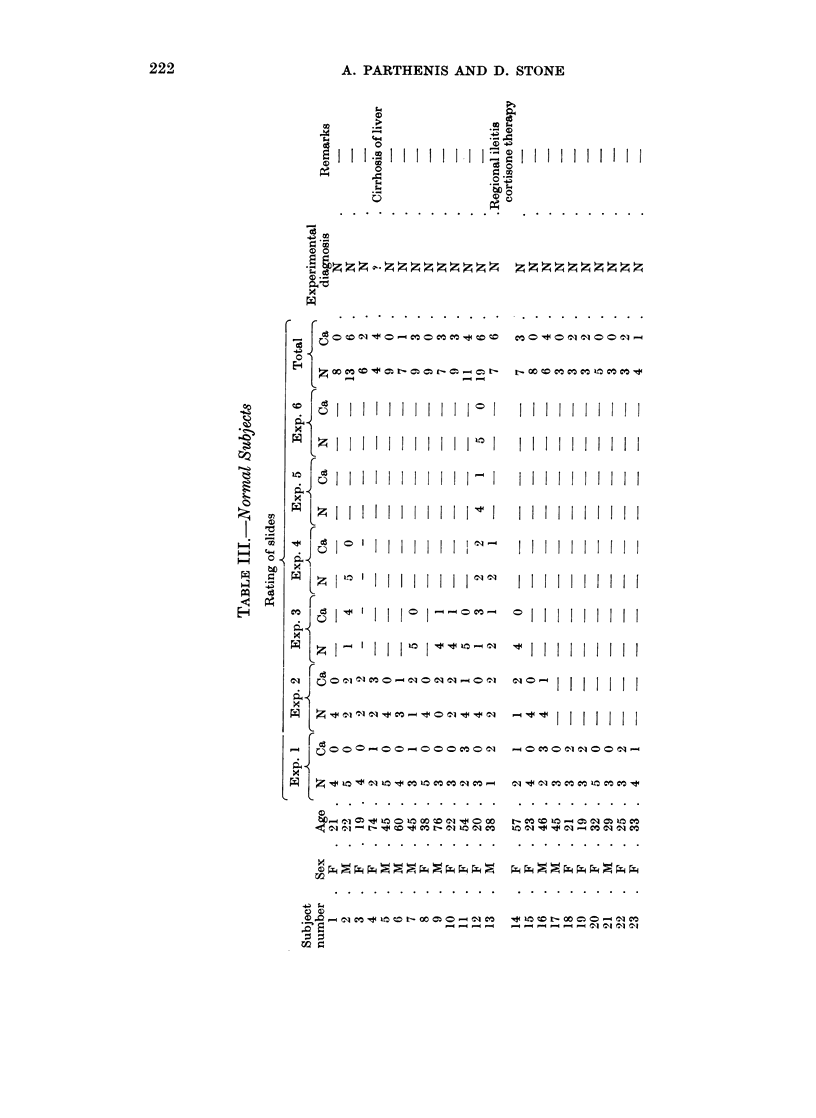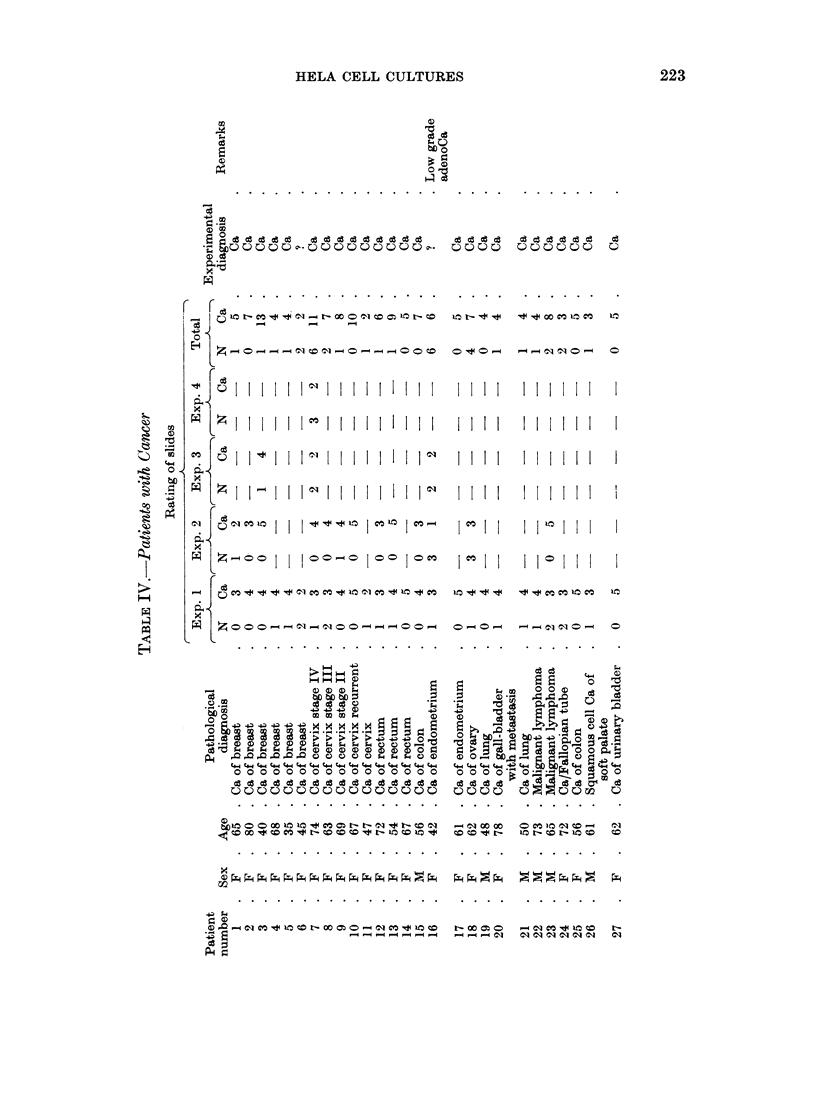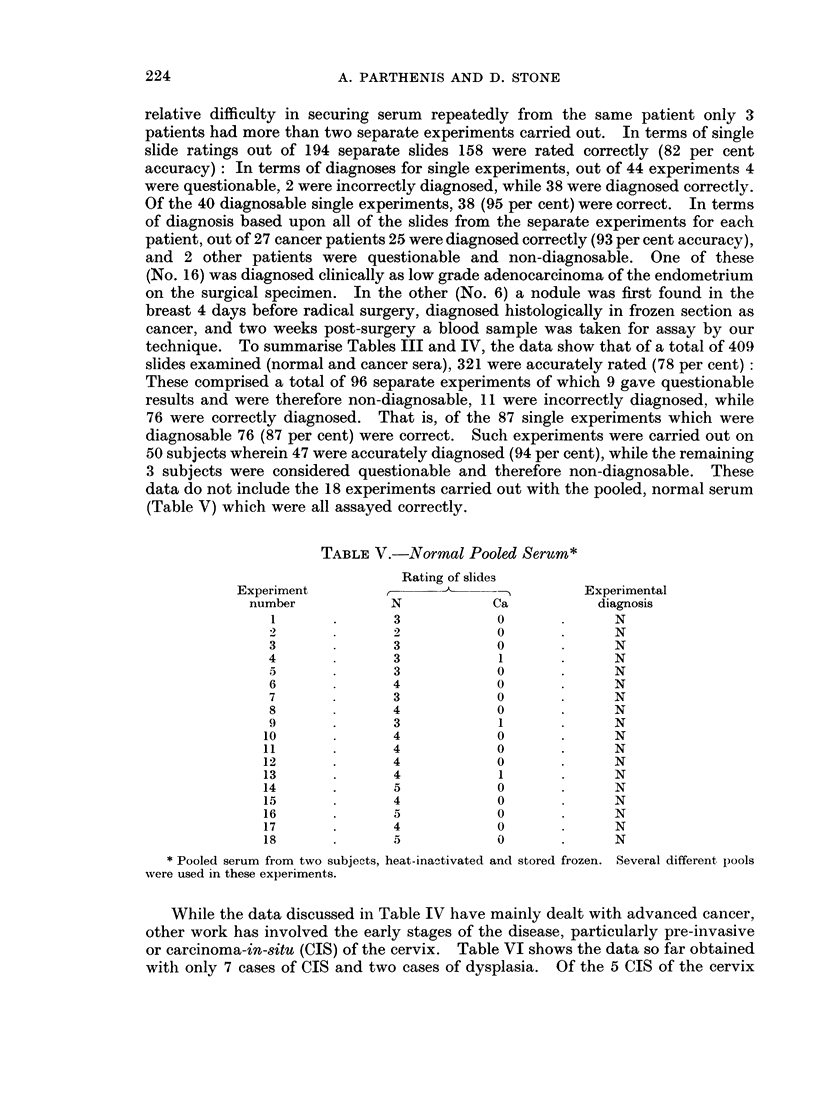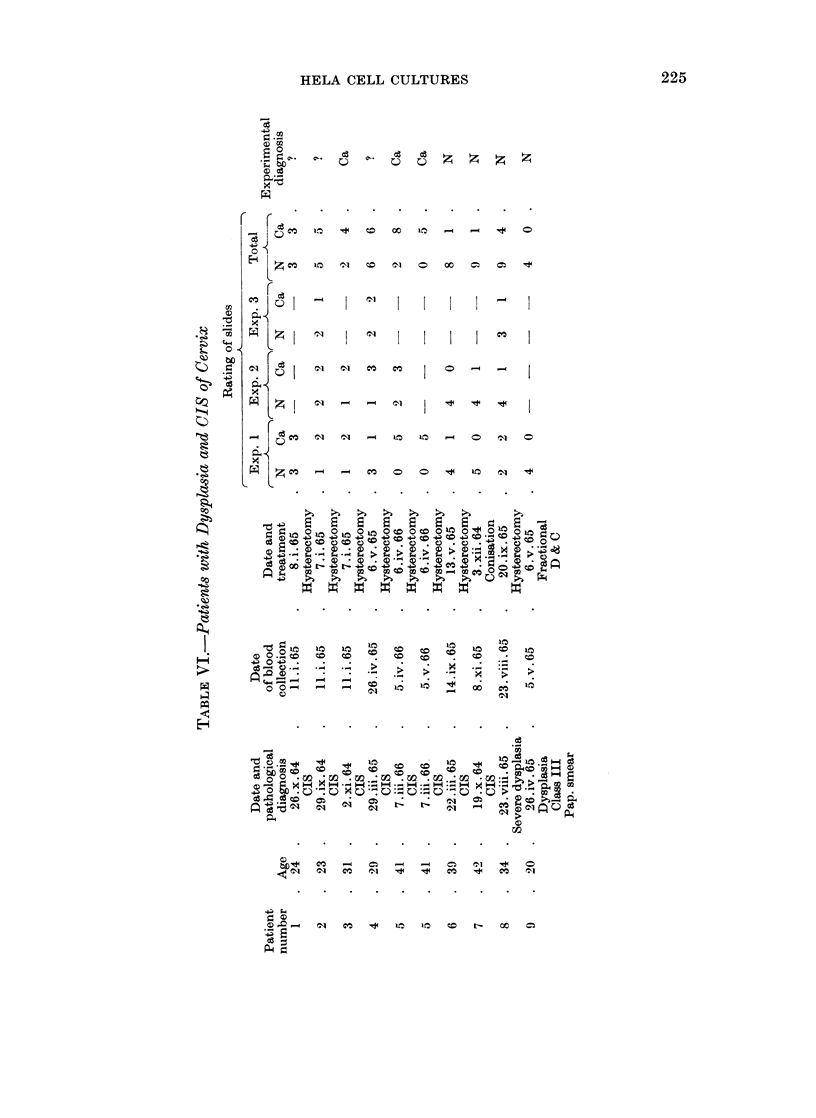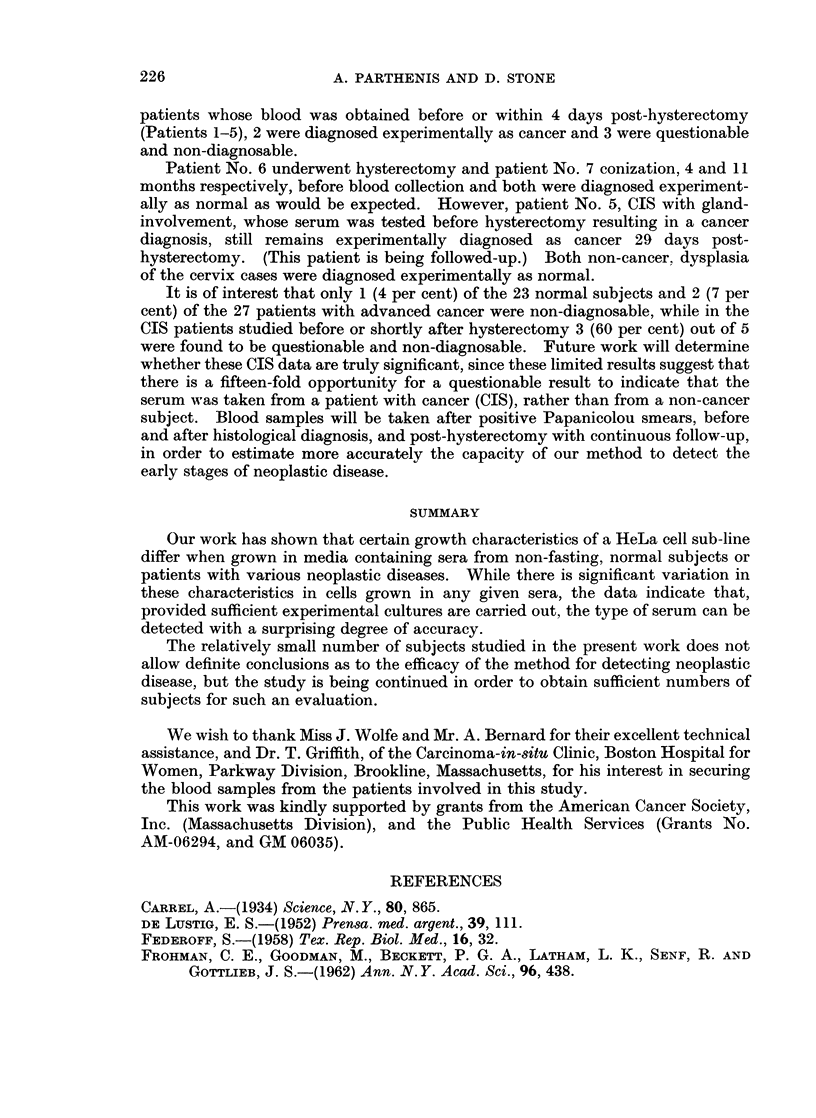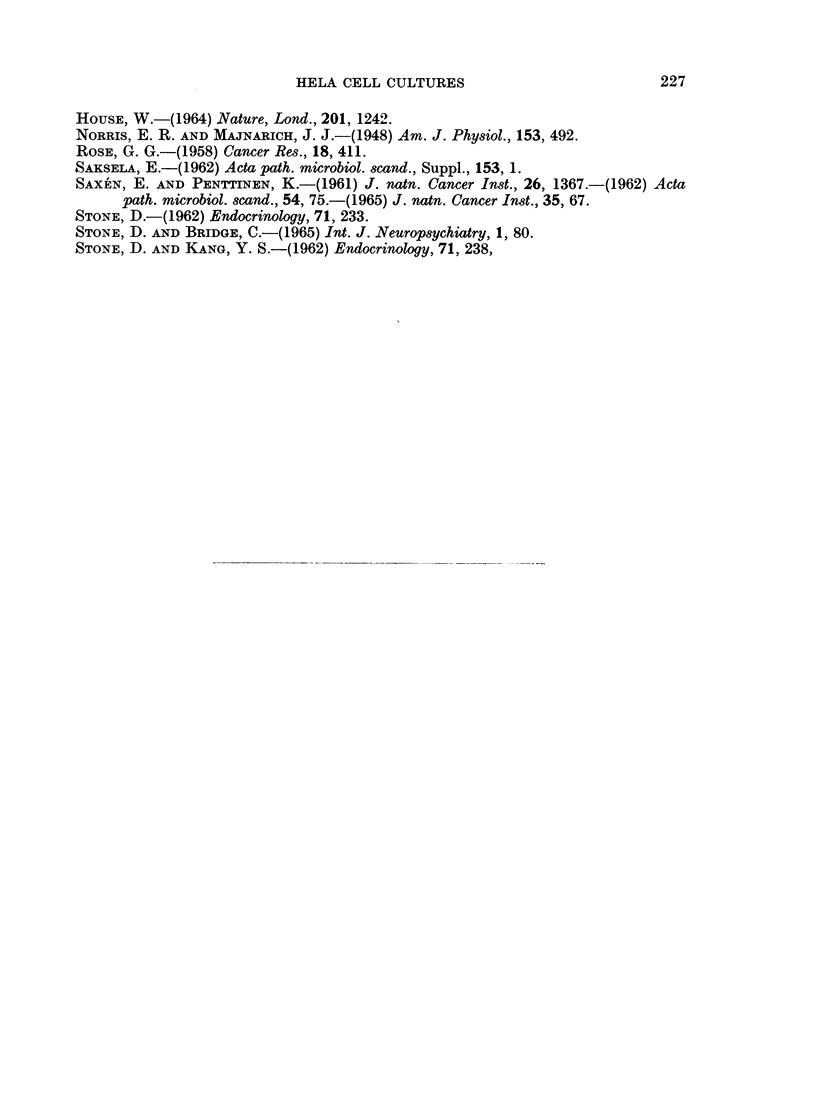# Some morphological differences in HeLa cell cultures when grown in the presence of normal human or cancer sera.

**DOI:** 10.1038/bjc.1967.24

**Published:** 1967-03

**Authors:** A. Parthenis, D. Stone

## Abstract

**Images:**


### SOME MORPHOLOGICAL DIFFERENCES IN HELA CELL CULTURES WHEN GROWN IN THE PRESENCE OF NORMAL HUMAN OR CANCER SERA

A. PARTHENIS AND D. STONE

*From the Worcester Foundation for Experimental Biology, Shrewsbury, Massachusetts and The Research Institute of Life Sciences of the Worcester State Hospital, Worcester, Massachusetts, U.S.A.*

Received for publication October 6, 1966

TISSUE culture represents a biological indicator for the properties of the surrounding medium (Carrel, 1934), and many papers have been published recording the different influences on cultured cells by growth-media supplemented with the sera from individual subjects, or between normal subjects and those with various pathological conditions. Fibroblast cultures have been shown to undergo mitotic stimulation in cancer serum, in comparison to inhibitory effects of normal serum (de Lustig, 1952). Cultures of chick erythrocytes are capable of detecting differences between normal and schizophrenic sera by differential effects on the carbohydrate metabolism of the cells (Frohman *et al.*, 1962), and, indeed, it has been reported (Stone and Bridge, 1965) that sera from normals and schizophrenic subjects appear to have different effects upon the chromosomal changes that take place in cultures of Chinese Hamster cells. Others have published on the effects of different human sera on the karyologic picture of HeLa cells (Saksela, 1962). As compared to serum from normal individuals, it has been reported that cancer serum markedly stimulates the appearance of variant pinocytic cells in the periphery of Hela cell colonies (Rose, 1958). Other investigators have observed that serum from cancer patients has growth-promoting effects (Norris and Majnarich, 1948), and work on the individual variability of the growth-controlling actions and cell-clumping effects of fresh human serum on several cell lines in culture, including HeLa cells, suggest relationships between the presence of certain lipoproteins and serum clumping (Saxén and Penttinen, 1961). Later work (Saxén and Penttinen, 1962) has indicated that fresh sera from cancer patients (and from women in late pregnancy) stimulated the growth of HeLa cell cultures, while sera from many patients of advanced age has a strong inhibitory action. On the basis of these studies (Saxén and Penttinen, 1962) it was suggested that in fresh human serum there are factors which can influence growth and cell-clumping ; and while variations in the activity of these factors depend upon the individual serum used, in cancer patients the distribution of clumping and non-clumping sera differed from that found in normal controls. A later publication by the same workers (Saxén and Penttinen, 1965) reported that two subjects whose serum had never shown clumping properties later did exhibit clumping activities, and in both subjects cancer was clinically diagnosed.

In the present study we have observed small but significant differences in cell cytology and colonial morphology of HeLa cells when they are grown in media containing normal human or cancer serum. In a series of double-blind experiments we have been able to distinguish between sera from normal subjects or patients with cancer with a relatively high degree of accuracy.

### METHODS

*Human sera.*—12–15 ml. of venous blood was collected by means of vacutainers (20 ml. capacity), and left to clot at room temperature for 15 to 20 minutes. It was then centrifuged for 15 minutes, and the serum transferred to 25 ml. glass bottles, and placed in a deep freeze. All subjects included in the study were non-fasting and were normal subjects randomly chosen, or had pathologically diagnosed, neoplastic disease. None of the patients with cancer were under chemotherapy ; three had X-ray treatment 5–7 days before blood collection.

*HeLa cells.*—HeLa cells obtained as sub-line HuE were maintained in 10 per cent heat-inactivated, pooled human serum and 90 per cent HeLa medium as previously published (Stone, 1962).

*Preparation of growth medium.*—The serum was allowed to thaw at room temperature, and 2 to 2·5 ml. were mixed with HeLa medium, containing penicillin and streptomycin, to make a 10 per cent serum concentration. Initially, filter-sterilization was carried out by gravity filtration through small asbestos filters, and occasionally, depending upon the volumes filtered, the media caused cell death. Similar toxicity has been reported by others (House, 1964). The use of Millipore filtration eliminated this problem.

*Inoculation and culture of HeLa cells.*—HeLa cells were harvested from stock Milk Dilution bottles by versene (Stone, 1962). Twenty thousand cells were inoculated into Leighton-tubes fitted with cover-slips and containing 1·5 ml. medium. Incubation was carried out at 38° C. The medium was changed on the 3rd day of culture, and on the 6th day the cover-slips were removed from the Leighton-tubes, fixed in Carnoy's and absolute alcohol, stained with aceto-carmine, and mounted with euparal on regular size slides.

*Experimental design.*—The experiments were carried out by employing a double-blind procedure. The technician, not the investigator, received the samples of normal and cancer sera, and included some or all of them in each experiment. Three to five Leighton tubes were used per subject in each of the experimental series, and the tubes were labelled by a code known only to the technician. Tubes containing 10 per cent heat-inactivated pooled-human serum were also used.

*Ratings.*—The rating of each slide as to which type of serum the cells had been grown was made by the investigator and based upon an evaluation of several morphological characteristics in various areas of the slide. Observations were made by light and phase microscopy.

Generally, cells cultured in serum from patients with cancer form a larger percentage of smaller colonies than do cells grown in normal serum. In the cancer medium there is a significantly greater tendency for the colonies, regardless of size, to exhibit a rounder, smoother periphery with more distinct borders, than is observed in normal medium (Fig. 1a). Under phase microscopy, in most areas of the slide, the nucleoplasm of cells grown in normal serum is stained darker than the cytoplasm, while in cancer medium there is a reversal, or a lack of contrast (Fig. 1b). Further, regardless of colony size, colonies formed in normal serum tend to be more homogeneous in terms of cell orientation and staining than those grown in cancer serum and, in most cases, the staining of the nucleus of the cell grown in cancer medium (Fig. 2b) is more heterogeneous than cells cultured in normal sera (Fig. 2a). Nuclear membranes are usually more distinct in the normal as compared to cancer serum (Fig. 2).

After the ratings were made the technician released the code to the investigator so that slides corresponding to each subject could be grouped. In any one experiment each subject could then be diagnosed as normal or cancer, depending upon the majority of normal or cancer ratings in each group of slides. The final diagnosis depended upon the majority of cancer or normal ratings for the total number of slides corresponding to each particular subject in several repeat experiments (see Tables of Results). Repeat experiments on individuals were carried out using either the same serum (stored for 3–40 days in the deep-freeze) or from blood collected on other occasions.

### RESULTS AND DISCUSSION

While other investigators have indicated growth stimulatory effects of sera from cancer patients as compared to normal subjects in supporting the growth of HeLa cells in culture, as shown in Table I, the cell line used in the present study generally behaves in the opposite manner, although significant variations in overall growth of the cells occur depending upon the particular normal or cancer serum used to supplement the growth medium. It should be noted, however, that while others have used fresh sera, in the present work the sera employed were frozen for periods varying from 3 to 40 days.

Moreover, it is well established that cells in culture can undergo morphological and metabolic changes as well as selection, and HeLa cell cultures in different laboratories may well represent alterations in the spectra of their metabolic activities. Indeed, from a single culture, HeLa cell sub-lines may be obtained by selection, which show different growth responses to steroid supplementation (Stone, 1962), as well as exhibit different chromosome numbers in their stem-cells (Stone and Kang, 1962). Serum from schizophrenics has been shown to be toxic to HeLa cells (Federoff, 1958), and in our laboratory we have had a HeLa sub-line which was completely inhibited in growth by the presence of serum from schizophrenic subjects, while the same serum did not exhibit such effect in other HeLa cultures (Stone and Bridge, 1965).

Our results are presented in the tables and show the number of subjects studied, their age, sex, pathological diagnosis, slide rating in each individual experiment, and final diagnosis from the total number of slides examined for each subject.

Table II summarises the final data obtained with 23 normal and 27 cancer subjects in stages more advanced than carcinoma-*in-situ*, and show that out of the 50 subjects, 47 were assayed correctly (i.e. 94 per cent accuracy). It will be noted that none of the experimental diagnoses was incorrect, but 1 of the normals

---

EXPLANATION OF PLATE.

FIG. 1.—Growth of HeLa cells in sera from normal subject and patient with cancer. Phase : ×150. (a) Normal serum. (b) Cancer serum.

FIG. 2.—Growth of HeLa cells in sera from normal subject and patient with cancer. Phase : ×380. (a) Normal serum. (b) Cancer serum.

1a 1b 2a 2b

Parthenis and Stone.

TABLE I.—*Growth of HeLa Cells in Medium Supplemented with Serum from Patients with Cancer as Compared to Growth in Serum from Normal Control Subjects*

| Patient number | Percentage of control growth* |
|---|---|
| 1 | 68 |
| 2 | 11 |
| 3 | 77 |
| 4 | 88 |
| 5 | 79 |
| 6 | 104 |
| 7 | 45 |
| Average growth | 67 |

* Cell numbers after 7 days of growth.

TABLE II.—*Summary of Results. Normal and Cancer Subjects More Advanced than Carcinoma-in-situ*

| Number of subjects studied | Clinical diagnosis | Experimental diagnosis | | |
|---|---|---|---|---|
| | | Normal | Cancer | Questionable |
| 23 | Normal | 22 | 0 | 1 |
| 27 | Cancer | 0 | 25 | 2 |
| 18 experiments | Normal* | 18 | 0 | 0 |

* Various pools of heat-inactivated serum, each from two normal subjects.

(4 per cent) of 2 of the cancer patients (7 per cent) each gave equal numbers of cancer and normal ratings, making the final experimental diagnosis for these particular subjects questionable. The table also shows that in an additional 18 experiments, pooled, heat-inactivated, normal human serum was used, and all experimental diagnoses were correct.

Table III shows the more detailed data for the normal subjects (14 female, 9 male, with ages varying from 19–76) in that the results of the individual experiments, as well as the final diagnoses from the total number of slides rated, are indicated. It will be seen that single experiments were carried out on 7 subjects, using 3 to 5 tubes in each, 8 subjects had duplicate experiments, each employing 4–5 tubes, and 3 or more experiments were carried out on 8 subjects.

With the exception of subject No. 13, repeats were based on random selection by the technician, or according to the availability of serum. (In the case of No. 13, the subject has had regional ileitis treated by partial resection of ileum and ascending colon, and since then has been on cortisone therapy.) In terms of single slide ratings, out of 215 slides 163 were rated correctly (76 per cent accuracy): In terms of diagnosis for single experiments, out of 52 experiments 5 show equal numbers of cancer and normal ratings and were, therefore, questionable and non-diagnosable, 9 were incorrectly diagnosed, and 38 were diagnosed correctly. Therefore, of the 47 diagnosable single experiments 38 (81 per cent) were correct. In terms of the final subject diagnosis based upon all of the slides rated for each subject, out of the 23 subjects 22 were diagnosed correctly (96 per cent accuracy). The remaining subject, with cirrhosis of the liver, showed 4 tubes rated as normal and 4 tubes as cancer, and was, therefore, considered questionable and non-diagnosable.

Table IV shows similar data for 27 patients (21 female, 6 male, with ages ranging from 35–80 years) with various neoplastic diseases. Because of the

TABLE III.—*Normal Subjects*

| Subject number | Sex | Age | Exp. 1 N | Exp. 1 Ca | Exp. 2 N | Exp. 2 Ca | Exp. 3 N | Exp. 3 Ca | Exp. 4 N | Exp. 4 Ca | Exp. 5 N | Exp. 5 Ca | Exp. 6 N | Exp. 6 Ca | Total N | Total Ca | Experimental diagnosis | Remarks |
|---|---|---|---|---|---|---|---|---|---|---|---|---|---|---|---|---|---|---|
| 1 | F | 21 | 4 | 0 | 4 | 0 | — | — | — | — | — | — | — | — | 8 | 0 | N | — |
| 2 | M | 22 | 5 | 0 | 2 | 2 | 1 | 4 | 5 | 0 | — | — | — | — | 13 | 6 | N | — |
| 3 | F | 19 | 4 | 0 | 2 | 2 | — | — | — | — | — | — | — | — | 6 | 2 | N | — |
| 4 | F | 74 | 2 | 1 | 2 | 3 | — | — | — | — | — | — | — | — | 4 | 4 | ? | Cirrhosis of liver |
| 5 | M | 45 | 5 | 0 | 4 | 0 | — | — | — | — | — | — | — | — | 9 | 0 | N | — |
| 6 | M | 60 | 4 | 0 | 3 | 1 | — | — | — | — | — | — | — | — | 7 | 1 | N | — |
| 7 | M | 45 | 3 | 1 | 1 | 2 | 5 | 0 | — | — | — | — | — | — | 9 | 3 | N | — |
| 8 | F | 38 | 5 | 0 | 4 | 0 | — | — | — | — | — | — | — | — | 9 | 0 | N | — |
| 9 | M | 76 | 3 | 0 | 0 | 2 | 4 | 1 | — | — | — | — | — | — | 7 | 3 | N | — |
| 10 | F | 22 | 3 | 0 | 2 | 2 | 4 | 1 | — | — | — | — | — | — | 9 | 3 | N | — |
| 11 | F | 54 | 2 | 3 | 4 | 1 | 5 | 0 | — | — | — | — | — | — | 11 | 4 | N | — |
| 12 | F | 20 | 3 | 0 | 4 | 0 | 1 | 3 | 2 | 2 | 4 | 1 | 5 | 0 | 19 | 6 | N | — |
| 13 | M | 38 | 1 | 2 | 2 | 2 | 2 | 1 | 2 | 1 | — | — | — | — | 7 | 6 | N | Regional ileitis cortisone therapy |
| 14 | F | 57 | 2 | 1 | 1 | 2 | 4 | 0 | — | — | — | — | — | — | 7 | 3 | N | — |
| 15 | F | 23 | 4 | 0 | 4 | 0 | — | — | — | — | — | — | — | — | 8 | 0 | N | — |
| 16 | M | 46 | 2 | 3 | 4 | 1 | — | — | — | — | — | — | — | — | 6 | 4 | N | — |
| 17 | M | 45 | 3 | 0 | — | — | — | — | — | — | — | — | — | — | 3 | 0 | N | — |
| 18 | F | 21 | 3 | 2 | — | — | — | — | — | — | — | — | — | — | 3 | 2 | N | — |
| 19 | F | 19 | 3 | 2 | — | — | — | — | — | — | — | — | — | — | 3 | 2 | N | — |
| 20 | F | 32 | 5 | 0 | — | — | — | — | — | — | — | — | — | — | 5 | 0 | N | — |
| 21 | M | 29 | 3 | 0 | — | — | — | — | — | — | — | — | — | — | 3 | 0 | N | — |
| 22 | F | 25 | 3 | 2 | — | — | — | — | — | — | — | — | — | — | 3 | 2 | N | — |
| 23 | F | 33 | 4 | 1 | — | — | — | — | — | — | — | — | — | — | 4 | 1 | N | — |

TABLE IV.—*Patients with Cancer*

| Patient number | Sex | Age | Pathological diagnosis | Rating of slides: Exp. 1 N | Exp. 1 Ca | Exp. 2 N | Exp. 2 Ca | Exp. 3 N | Exp. 3 Ca | Exp. 4 N | Exp. 4 Ca | Total N | Total Ca | Experimental diagnosis | Remarks |
|---|---|---|---|---|---|---|---|---|---|---|---|---|---|---|---|
| 1 | F | 65 | Ca of breast | 0 | 3 | 1 | 2 | — | — | — | — | 1 | 5 | Ca | |
| 2 | F | 80 | Ca of breast | 0 | 4 | 0 | 3 | — | — | — | — | 0 | 7 | Ca | |
| 3 | F | 40 | Ca of breast | 0 | 4 | 0 | 5 | 1 | 4 | — | — | 1 | 13 | Ca | |
| 4 | F | 68 | Ca of breast | 1 | 4 | — | — | — | — | — | — | 1 | 4 | Ca | |
| 5 | F | 35 | Ca of breast | 1 | 4 | — | — | — | — | — | — | 1 | 4 | Ca | |
| 6 | F | 45 | Ca of breast | 2 | 2 | — | — | — | — | — | — | 2 | 2 | ? | |
| 7 | F | 74 | Ca of cervix stage IV | 1 | 3 | 0 | 4 | 2 | 2 | 3 | 2 | 6 | 11 | Ca | |
| 8 | F | 63 | Ca of cervix stage III | 2 | 3 | 0 | 4 | — | — | — | — | 2 | 7 | Ca | |
| 9 | F | 69 | Ca of cervix stage II | 0 | 4 | 1 | 4 | — | — | — | — | 1 | 8 | Ca | |
| 10 | F | 67 | Ca of cervix recurrent | 0 | 5 | 0 | 5 | — | — | — | — | 0 | 10 | Ca | |
| 11 | F | 47 | Ca of cervix | 1 | 2 | — | — | — | — | — | — | 1 | 2 | Ca | |
| 12 | F | 72 | Ca of rectum | 1 | 3 | 0 | 3 | — | — | — | — | 1 | 6 | Ca | |
| 13 | F | 54 | Ca of rectum | 1 | 4 | 0 | 5 | — | — | — | — | 1 | 9 | Ca | |
| 14 | F | 67 | Ca of rectum | 0 | 5 | — | — | — | — | — | — | 0 | 5 | Ca | |
| 15 | M | 56 | Ca of colon | 0 | 4 | 0 | 3 | — | — | — | — | 0 | 7 | Ca | |
| 16 | F | 42 | Ca of endometrium | 1 | 3 | 3 | 1 | 2 | 2 | — | — | 6 | 6 | ? | Low grade adenoCa |
| 17 | F | 61 | Ca of endometrium | 0 | 5 | — | — | — | — | — | — | 0 | 5 | Ca | |
| 18 | F | 62 | Ca of ovary | 1 | 4 | 3 | 3 | — | — | — | — | 4 | 7 | Ca | |
| 19 | M | 48 | Ca of lung | 0 | 4 | — | — | — | — | — | — | 0 | 4 | Ca | |
| 20 | F | 78 | Ca of gall-bladder with metastasis | 1 | 4 | — | — | — | — | — | — | 1 | 4 | Ca | |
| 21 | M | 50 | Ca of lung | 1 | 4 | — | — | — | — | — | — | 1 | 4 | Ca | |
| 22 | M | 73 | Malignant lymphoma | 1 | 4 | — | — | — | — | — | — | 1 | 4 | Ca | |
| 23 | M | 65 | Malignant lymphoma | 2 | 3 | 0 | 5 | — | — | — | — | 2 | 8 | Ca | |
| 24 | F | 72 | Ca/Fallopian tube | 2 | 3 | — | — | — | — | — | — | 2 | 3 | Ca | |
| 25 | F | 56 | Ca of colon | 0 | 5 | — | — | — | — | — | — | 0 | 5 | Ca | |
| 26 | M | 61 | Squamous cell Ca of soft palate | 1 | 3 | — | — | — | — | — | — | 1 | 3 | Ca | |
| 27 | F | 62 | Ca of urinary bladder | 0 | 5 | — | — | — | — | — | — | 0 | 5 | Ca | |

relative difficulty in securing serum repeatedly from the same patient only 3 patients had more than two separate experiments carried out. In terms of single slide ratings out of 194 separate slides 158 were rated correctly (82 per cent accuracy) : In terms of diagnoses for single experiments, out of 44 experiments 4 were questionable, 2 were incorrectly diagnosed, while 38 were diagnosed correctly. Of the 40 diagnosable single experiments, 38 (95 per cent) were correct. In terms of diagnosis based upon all of the slides from the separate experiments for each patient, out of 27 cancer patients 25 were diagnosed correctly (93 per cent accuracy), and 2 other patients were questionable and non-diagnosable. One of these (No. 16) was diagnosed clinically as low grade adenocarcinoma of the endometrium on the surgical specimen. In the other (No. 6) a nodule was first found in the breast 4 days before radical surgery, diagnosed histologically in frozen section as cancer, and two weeks post-surgery a blood sample was taken for assay by our technique. To summarise Tables III and IV, the data show that of a total of 409 slides examined (normal and cancer sera), 321 were accurately rated (78 per cent) : These comprised a total of 96 separate experiments of which 9 gave questionable results and were therefore non-diagnosable, 11 were incorrectly diagnosed, while 76 were correctly diagnosed. That is, of the 87 single experiments which were diagnosable 76 (87 per cent) were correct. Such experiments were carried out on 50 subjects wherein 47 were accurately diagnosed (94 per cent), while the remaining 3 subjects were considered questionable and therefore non-diagnosable. These data do not include the 18 experiments carried out with the pooled, normal serum (Table V) which were all assayed correctly.

TABLE V.—*Normal Pooled Serum**

| Experiment number | Rating of slides N | Rating of slides Ca | Experimental diagnosis |
|---|---|---|---|
| 1 | 3 | 0 | N |
| 2 | 2 | 0 | N |
| 3 | 3 | 0 | N |
| 4 | 3 | 1 | N |
| 5 | 3 | 0 | N |
| 6 | 4 | 0 | N |
| 7 | 3 | 0 | N |
| 8 | 4 | 0 | N |
| 9 | 3 | 1 | N |
| 10 | 4 | 0 | N |
| 11 | 4 | 0 | N |
| 12 | 4 | 0 | N |
| 13 | 4 | 1 | N |
| 14 | 5 | 0 | N |
| 15 | 4 | 0 | N |
| 16 | 5 | 0 | N |
| 17 | 4 | 0 | N |
| 18 | 5 | 0 | N |

* Pooled serum from two subjects, heat-inactivated and stored frozen. Several different pools were used in these experiments.

While the data discussed in Table IV have mainly dealt with advanced cancer, other work has involved the early stages of the disease, particularly pre-invasive or carcinoma-*in-situ* (CIS) of the cervix. Table VI shows the data so far obtained with only 7 cases of CIS and two cases of dysplasia. Of the 5 CIS of the cervix

TABLE VI.—*Patients with Dysplasia and CIS of Cervix*

| Patient number | Age | Date and pathological diagnosis | Date of blood collection | Date and treatment | Rating of slides: Exp. 1 N | Exp. 1 Ca | Exp. 2 N | Exp. 2 Ca | Exp. 3 N | Exp. 3 Ca | Total N | Total Ca | Experimental diagnosis |
|---|---|---|---|---|---|---|---|---|---|---|---|---|---|
| 1 | 24 | 26.x.64 CIS | 11.i.65 | 8.i.65 Hysterectomy | 3 | 3 | — | — | — | — | 3 | 3 | ? |
| 2 | 23 | 29.ix.64 CIS | 11.i.65 | 7.i.65 Hysterectomy | 1 | 2 | 2 | 2 | 2 | 1 | 5 | 5 | ? |
| 3 | 31 | 2.xi.64 CIS | 11.i.65 | 7.i.65 Hysterectomy | 1 | 2 | 1 | 2 | — | — | 2 | 4 | Ca |
| 4 | 29 | 29.iii.65 CIS | 26.iv.65 | 6.v.65 Hysterectomy | 3 | 1 | 1 | 3 | 2 | 2 | 6 | 6 | ? |
| 5 | 41 | 7.iii.66 CIS | 5.iv.66 | 6.iv.66 Hysterectomy | 0 | 5 | 2 | 3 | — | — | 2 | 8 | Ca |
| 5 | 41 | 7.iii.66 CIS | 5.v.66 | 6.iv.66 Hysterectomy | 0 | 5 | — | — | — | — | 0 | 5 | Ca |
| 6 | 39 | 22.iii.65 CIS | 14.ix.65 | 13.v.65 Hysterectomy | 4 | 1 | 4 | 0 | — | — | 8 | 1 | N |
| 7 | 42 | 19.x.64 CIS | 8.xi.65 | 3.xii.64 Conisation | 5 | 0 | 4 | 1 | — | — | 9 | 1 | N |
| 8 | 34 | 23.viii.65 Severe dysplasia | 23.viii.65 | 20.ix.65 Hysterectomy | 2 | 2 | 4 | 1 | 3 | 1 | 9 | 4 | N |
| 9 | 20 | 26.iv.65 Dysplasia Class III Pap. smear | 5.v.65 | 6.v.65 Fractional D & C | 4 | 0 | — | — | — | — | 4 | 0 | N |

patients whose blood was obtained before or within 4 days post-hysterectomy (Patients 1–5), 2 were diagnosed experimentally as cancer and 3 were questionable and non-diagnosable.

Patient No. 6 underwent hysterectomy and patient No. 7 conization, 4 and 11 months respectively, before blood collection and both were diagnosed experimentally as normal as would be expected. However, patient No. 5, CIS with gland-involvement, whose serum was tested before hysterectomy resulting in a cancer diagnosis, still remains experimentally diagnosed as cancer 29 days post-hysterectomy. (This patient is being followed-up.) Both non-cancer, dysplasia of the cervix cases were diagnosed experimentally as normal.

It is of interest that only 1 (4 per cent) of the 23 normal subjects and 2 (7 per cent) of the 27 patients with advanced cancer were non-diagnosable, while in the CIS patients studied before or shortly after hysterectomy 3 (60 per cent) out of 5 were found to be questionable and non-diagnosable. Future work will determine whether these CIS data are truly significant, since these limited results suggest that there is a fifteen-fold opportunity for a questionable result to indicate that the serum was taken from a patient with cancer (CIS), rather than from a non-cancer subject. Blood samples will be taken after positive Papanicolou smears, before and after histological diagnosis, and post-hysterectomy with continuous follow-up, in order to estimate more accurately the capacity of our method to detect the early stages of neoplastic disease.

### SUMMARY

Our work has shown that certain growth characteristics of a HeLa cell sub-line differ when grown in media containing sera from non-fasting, normal subjects or patients with various neoplastic diseases. While there is significant variation in these characteristics in cells grown in any given sera, the data indicate that, provided sufficient experimental cultures are carried out, the type of serum can be detected with a surprising degree of accuracy.

The relatively small number of subjects studied in the present work does not allow definite conclusions as to the efficacy of the method for detecting neoplastic disease, but the study is being continued in order to obtain sufficient numbers of subjects for such an evaluation.

We wish to thank Miss J. Wolfe and Mr. A. Bernard for their excellent technical assistance, and Dr. T. Griffith, of the Carcinoma-*in-situ* Clinic, Boston Hospital for Women, Parkway Division, Brookline, Massachusetts, for his interest in securing the blood samples from the patients involved in this study.

This work was kindly supported by grants from the American Cancer Society, Inc. (Massachusetts Division), and the Public Health Services (Grants No. AM-06294, and GM 06035).

### REFERENCES

CARREL, A.—(1934) *Science, N.Y.*, **80**, 865.

DE LUSTIG, E. S.—(1952) *Prensa. med. argent.*, **39**, 111.

FEDEROFF, S.—(1958) *Tex. Rep. Biol. Med.*, **16**, 32.

FROHMAN, C. E., GOODMAN, M., BECKETT, P. G. A., LATHAM, L. K., SENF, R. AND GOTTLIEB, J. S.—(1962) *Ann. N.Y. Acad. Sci.*, **96**, 438.

HOUSE, W.—(1964) *Nature, Lond.*, **201**, 1242.
NORRIS, E. R. AND MAJNARICH, J. J.—(1948) *Am. J. Physiol.*, **153**, 492.
ROSE, G. G.—(1958) *Cancer Res.*, **18**, 411.
SAKSELA, E.—(1962) *Acta path. microbiol. scand.*, Suppl., **153**, 1.
SAXÉN, E. AND PENTTINEN, K.—(1961) *J. natn. Cancer Inst.*, **26**, 1367.—(1962) *Acta path. microbiol. scand.*, **54**, 75.—(1965) *J. natn. Cancer Inst.*, **35**, 67.
STONE, D.—(1962) *Endocrinology*, **71**, 233.
STONE, D. AND BRIDGE, C.—(1965) *Int. J. Neuropsychiatry*, **1**, 80.
STONE, D. AND KANG, Y. S.—(1962) *Endocrinology*, **71**, 238,